# The WSES: what do we see in the future?

**DOI:** 10.1186/s13017-021-00358-z

**Published:** 2021-03-20

**Authors:** Belinda De Simone, Yoram Kluger, Ernest E. Moore, Salomone Di Saverio, Massimo Sartelli, Luca Ansaloni, Federico Coccolini, Walter L. Biffl, Fausto Catena

**Affiliations:** 1 Department of General, Emergency and Metabolic Minimally Invasive Surgery, Poissy and Saint Germain en Laye Hospitals, 10 rue du Champ Gaillard, 78300 Poissy, France; 2 Department of Emergency and Trauma Surgery, Rambam Health Campus, Haifa, Israel; 3 Shock Trauma Center at Denver Health, Denver, CO USA; 4 Department of General Surgery, University Hospital of Varese, University of Insubria, Varese, Italy; 5 Department of General Surgery, Macerata Hospital, Macerata, Italy; 6 Department of General Surgery, University Hospital of Pavia, Pavia, Italy; 7 Department of Trauma and Emergency Surgery, University Hospital of Pisa, Pisa, Italy; 8 Department of Trauma and Acute Care Surgery, Scripps Memorial Hospital, La Jolla, CA USA; 9 Department of Emergency and Trauma Surgery, University Hospital of Parma, Parma, Italy

**Keywords:** Emergency surgery, WSES, Program, Inclusivity, Equality, Education, WJES, Artificial intelligence

## Abstract

We present the New Year letter from the WSES board to wish everyone a new year full of positive surprises and good news, despite COVID-19 pandemic.

We confirm the WSES primary aim: to promote education in emergency surgery putting together all the world experts on emergency surgery without restrictions or boundaries, in inclusivity, equality, and equal opportunities. This will be the year of innovations and WSES will assess the application of artificial intelligence technologies in emergency and trauma surgery.

Thank you All for trusting us with your collaboration.

## Main text

The year that just passed over was challenging.

The WSES was founded in 2007 to represent not another scientific society designed to give visibility to certain people or to compete with other societies or associations with an annual Congress, but to promote education in emergency surgery [1].

At the WSES Bergamo Congress in 2013, the WSES-WJES board established the Scientific Development Policy for the Society and Journal: the project was based on the idea to create an organized scientific movement having the objective to standardize the state of the art for emergency surgery while attempting to develop guidelines for related topics and promoting original research [2].

In these years, the society matured, including members from all over the world and the WJES reached an IF of 4.1. The WSES is the only international society to have conceived and developed the Congress Impact Factor to assess the scientific quality of local and international conventions to help physicians and surgeons in choosing the most appropriate conference, meeting or congress [3].

The WSES-WJES Educational Team organized international courses, published Trauma Surgery and Acute Care (non-trauma) books to promulgate emergency surgery education around the world, by using WSES-WJES guidelines.

High impact guidelines about hot topics in emergency surgery were conceived and published and now they represent an important tool to define the good clinical practice.

All these goals result from a friendly and inclusive collaboration among high expertise academic and hospital trauma and acute care surgeons of all nationality with *no restrictions or boundaries.*

The year 2020 that just passed over was challenging for all of us; we faced COVID-19 pandemic that has changed our vision of practice and life.

Every one of us such as surgeon and human being experienced isolation from beloved family and friends and empathy for all the healthcare professionals and people that died because SARS-CoV-2, but this did not demotivate WSES board members.

The first WSES Congress web has been held in November 2020.

We realized the importance of technologies in modern surgical education and practice, to shorten distances and go on with scientific activity and surgical education, inspiring young emergency surgeons.

This era of surgical education is characterized by rapid and dynamic changes in knowledge, understanding of the surgical disease, new procedures, and technologies to artificial intelligence.

The time is right to be social and progress in communication and diffusion of guidelines by:

- A YouTube channel to show new techniques and devices in trauma and emergency surgery;
- The WSES Academy webinars project.

Our main aims are again *inclusion, equality* and *equal opportunities*, involving as much as possible professionals working in different countries and continents to provide common tools to identify the best clinical practice.

We are probably the “last of the Mohicans”; a sort of special force of general surgery, selected and to be preserved, but we are not few and we claim the importance of emergency surgery [4].

This is a new start, a new beginning.

Happy New Year!

## Conclusion

This is the New Year letter from WSES board to wish everyone a new year full of positive surprises and good news, despite COVID-19 pandemic.

With a glance to the past experiences and goals fulfilled, we want to repeat that our primary objective is promoting education in emergency surgery in inclusivity, equality, and equal opportunities.

For the future, we plan to be more social, dynamic, and modern in communication and sharing our scientific activity.

This is a new start, a new beginning.

## Data Availability

Not applicable

## Abbreviations

WSES: World Society of Emergency Surgery
WJES: World Journal of Emergency Surgery
